# Linking freshwater ecotoxicity to damage on ecosystem services in life cycle assessment

**DOI:** 10.1016/j.envint.2022.107705

**Published:** 2023-01

**Authors:** Susan A. Oginah, Leo Posthuma, Lorraine Maltby, Michael Hauschild, Peter Fantke

**Affiliations:** a Quantitative Sustainability Assessment, Department of Environmental and Resource Engineering, Technical University of Denmark, Produktionstorvet 424, 2800 Kgs. Lyngby, Denmark; b National Institute for Public Health and the Environment, PO Box 1, 3720 Bilthoven, the Netherlands; c Department of Environmental Science, Radboud University Nijmegen, Heyendaalseweg, Nijmegen, the Netherlands; d School of Biosciences, The University of Sheffield, Sheffield S10 2TN, United Kingdom

**Keywords:** Species loss, Ecosystem functioning, Species diversity, Functional diversity, Chemical toxicity, Life cycle impact assessment

## Abstract

Freshwater ecosystems provide major benefits to human wellbeing—so-called ecosystem services (ES)—but are currently threatened among others by ecotoxicological pressure from chemicals reaching the environment. There is an increased motivation to incorporate ES in quantification tools that support decision-making, such as life cycle assessment (LCA). However, mechanistic models and frameworks that can systematically translate ecotoxicity effect data from chemical tests into eventual damage on species diversity, functional diversity, and ES in the field are still missing. While current approaches focus on translating predicted ecotoxicity impacts to damage in terms of species loss, no approaches are available in LCA and other comparative assessment frameworks for linking ecotoxicity to damage on ecosystem functioning or ES.

To overcome this challenge, we propose a way forward based on evaluating available approaches to characterize damage of chemical pollution on freshwater ES. We first outline an overall framework for linking freshwater ecotoxicity effects to damage on related ES in compliance with the boundary conditions of quantitative, comparative assessments. Second, within the proposed framework, we present possible approaches for stepwise linking ecotoxicity effects to species loss, functional diversity loss, and damage on ES. Finally, we discuss strengths, limitations, and data availability of possible approaches for each step.

Although most approaches for directly deriving damage on ES from either species loss or damage to functional diversity have not been operationalized, there are some promising ways forward. The Threshold Indicator Taxa ANalysis (TITAN) seems suitable to translate predicted ecotoxicity effects to a metric of quantitative damage on species diversity. A Trait Probability Density Framework (TPD) approach that incorporates various functional diversity components and functional groups could be adapted to link species loss to functional diversity loss. An Ecological Production Function (EPF) approach seems most promising for further linking functional diversity loss to damage on ES flows for human wellbeing. However, in order to integrate the entire pathway from predicted freshwater ecotoxicity to damage on ES into LCA and other comparative frameworks, the approaches adopted for each step need to be harmonized in terms of assumptions, boundary conditions and consistent interfaces with each other.

## Graphical abstract

**Figure:**
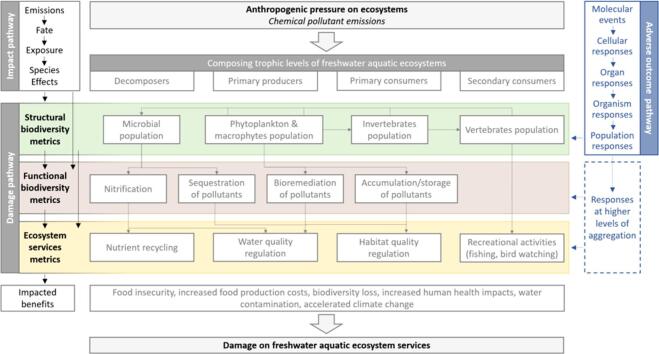

## 1 Introduction

Aquatic ecosystems provide essential benefits to our global society and human wellbeing (UNEP, 2017). These benefits are collectively known as ecosystem services (ES) (Awuah et al., 2020, Faber et al., 2019). Obvious ES that are provided by freshwater ecosystems mainly relate to the provisioning of food and drinking water, cultural services, recreational fishing, and ecotourism (Banerjee et al., 2013, Syberg et al., 2017, UNEP, 2017). Other benefits, such as maintaining habitat quality, water quality regulation through organic matter degradation and toxicant removal, and nutrient recycling, are less obvious yet essential for a sustainable development (UNEP, 2017).

Despite these benefits, freshwater ecosystems face continuously increasing pressures from human activities, such as pollution from chemicals emitted along product life cycles (Carney Almroth et al., 2022, Kosnik et al., 2022, Persson et al., 2022, Syberg et al., 2017), which interfere with species diversity and the ecosystem functions depending on those (Awuah et al., 2020), both of which are essential for providing ES. More specifically, chemical pollution from human activities and its pressure on aquatic ecosystems has been listed as a driving factor limiting maintenance of the desired ecological and chemical status of freshwater ecosystems worldwide (Posthuma et al., 2020, Millennium Ecosystem Assessment, 2005). Such pressure mainly occurs through interference with ecosystem structure (i.e. species abundances and species assemblage composition) and functions (e.g. dynamic food webs) (Maltby et al., 2017a, Maltby et al., 2017b). Chemical pollution pressure on freshwater ecosystems does not only have a direct impact on aquatic species (referred to as services providing units (SPU) in the context of ES) but also reduces their capacity to generate ES in ways that negatively impact human wellbeing, thus constituting a threat to sustainable ES production (Awuah et al., 2020, Liu et al., 2020).

Several authors have considered how to incorporate protecting or restoring ES in decision-making (Daily et al., 2009, Faber et al., 2019, Faber et al., 2021, Maltby et al., 2021), which requires knowledge of the characteristics and interlinkages of ES as well as tools that enable quantifying and evaluating ES (Maia de Souza et al., 2018). This requires an assessment along the source-to-damage pathway from evaluating the pressures, relating pressures to impacts on aquatic ecosystems (fate-exposure-effect chains), and translating these impacts into damage (referred to as damage on a defined environmental area of protection, such as ecosystem quality) caused to ecosystem structure (species abundance change/species loss/species diversity loss), damage on ecosystem functioning (functional diversity loss), and finally damage on relevant, interconnected ES.

Quantitative decision support tools, such as life cycle assessment (LCA), chemical substitution or chemical footprinting, have been developed in support of assessing and increasing environmental sustainability of products and technologies (Fantke and Illner, 2019, Koellner and Geyer, 2013, Liu et al., 2020, Othoniel et al., 2015. Such tools are generally designed to quantify the pathways from pressures to damages on ecosystems (Woods et al., 2018), which also includes the ecotoxicity impact pathway associated with chemical emissions along product life cycles (Fantke et al., 2018, Henderson et al., 2011, Westh et al., 2015). Ecotoxicity impact characterization is part of the life cycle impact assessment (LCIA) phase of LCA, and a recognized element of e.g. the European Product Environmental Footprint (PEF) approach for comparative evaluation of product-related footprints (Fantke et al., 2018, Saouter et al., 2017a, Saouter et al., 2017b).

The translation of predicted ecotoxicity impacts into aquatic species loss as LCA-metric for damage on ecosystem quality remains challenging, given the large diversity of chemical compounds, the required step to extrapolate from ecotoxicity test data to predicted toxic pressure, the largely unresolved association between the predicted toxic pressure and structural or functional damage in terms of, for example, species loss and altered food web function in the field, and the location-dependent variation in many parameters that influence the outcome of the impact pathway from emissions to change in ecosystem services. For the purpose of comparative LCA, the mechanistic or empirical association between insights from laboratory test data and eventual damage in the field is of interest, given the principle that other impact pathways also aim to characterize damage in the same units. Considering this pathway for chemical pollution highlights various challenges related to disconnects between current approaches and final damage aspects. There may be, for example, within-ecosystem species shifts as function of chemical pressure that would not lead to net species loss or significant functional damage (Liess et al., 2021).

Damage on ecosystem functioning or even further on ES associated with ecotoxicity impacts are currently not addressed in LCA. This is despite the fact that inclusion of ES in LCA to assess the importance and magnitude of different stressors on ecosystems and their respective services is the focus of several ongoing research efforts (Liu et al., 2020, Maia de Souza et al., 2018, Othoniel et al., 2015, Rugani et al., 2019). Among these efforts, Othoniel et al. (2015) identified challenges in emerging approaches for addressing ES in LCA, which include insufficient knowledge on spatiotemporal aspects and uncertainty in aggregating LCA indicator scores, and which does not reflect differences in damage levels across ES. They suggested that LCIA modelling of ES could benefit when harmonized with existing, integrated multiscale dynamic ES approaches (Othoniel et al., 2015, Maia de Souza et al., 2018).

In another study, Maia de Souza et al. (2018) discuss gaps and potential solutions for integrating ES assessment more broadly into the LCA framework. They propose that tools relying on extrapolation of ecosystems' functional production to their ES, such as the 'Integrated Valuation of Ecosystem Services and Tradeoffs' (InVEST) or the 'Multiscale Integrated Model of Ecosystem Services' (MIMES), might be useful to address the nonlinear nature of ES responses to pressures. Furthermore, they propose that applying ecosystem classification frameworks, such as the 'Common International Classification of Ecosystem Services' (CICES) or the 'National Ecosystem Services Classification System' (NESCS) or the 'Final Ecosystem Goods and Services Classification System' (FEGS-CS), can be relevant starting points to evaluate impacts from an ecosystem functional level up to damage on human wellbeing via ES. While such tools and classification systems seem to be useful for generally addressing ES in LCA, their applicability to ecotoxicity-related damage on ES is currently unclear. Rugani et al., 2019, Liu et al., 2020 propose a cascade framework that generally links changes in ecosystem structure and functions to changes in human wellbeing, and that aligns with the LCA cause effect chain model. This cascade framework is based on earlier work by Haines-Young and Potschin (2013), which links the flow of different ES from the source to their value for human wellbeing (Maia de Souza et al., 2018). In this cascade framework, again, ecotoxicity-related aspects and their influence on aquatic ES are not currently considered.

An approach that was discussed for overcoming the complexity of assessing ES, which could also be potentially useful in the context of LCA, is the use of ecological production functions (EPFs) to quantify and predict changes between specific ecosystem functions and ES (Bruins et al., 2017, Faber et al., 2021, Othoniel et al., 2015), by linking to changes in the characteristics and performance of service providing units (SPU), such as biomass, species richness or functional traits. However, various links from ecotoxicity impacts to damage on ES remain unaddressed or face significant data gaps.

In all, despite some emerging concepts to generally evaluate ES in LCA, challenges for including freshwater ES associated with ecotoxicity impacts from chemical life cycle emissions would be valuable for decision support, though remain largely unresolved. In order to quantify ecotoxicity-related damage on services provided by freshwater ecosystems, the main human-valued ES need to be first defined, including their underlying pathways from pressures to species and functional diversity loss in freshwater ecosystems, and finally to damage on ES. The present study aims at addressing this knowledge gap and proposes a way forward to characterize damage of chemical pollution on ES of freshwater ecosystems in LCA. This is done by focusing on three specific objectives: (a) to outline an overall framework for linking predicted freshwater ecotoxicity impacts to damage on related ES in compliance with the boundary conditions of LCA; (b) to present possible approaches for linking predicted ecotoxicity impacts to species loss and functional diversity loss, and finally to damage on ES in LCA; and (c) to discuss strengths, limitations and data availability of possible approaches for each step from ecotoxicity impacts to damage on ES.

## 2 Conceptual framework to link chemical emissions to damage on ecosystem services

Linking chemical emissions via predicted ecotoxicity impacts to damage on ES is not straightforward. When developing the pathway from ecotoxicity impacts to damage on ES, the main link is often from predicted species-level effects to damage on structural biodiversity (in the context of LCA typically referred to as species diversity or species loss), further to damage on functional (bio-)diversity loss, and finally to damage on related ES (Truchy et al., 2015, Maltby et al., 2021). Alternatively, there is the option to derive a direct link from species loss to damage on ES, without considering the intermediate step of evaluating impacts on any ecological function (Maltby et al., 2021). Further, ecosystem functioning can change without species loss (i.e., due to behavioural change), so that damage to ES may follow directly from such ecotoxicity effects (Truchy et al., 2015).

**Fig. 1:**
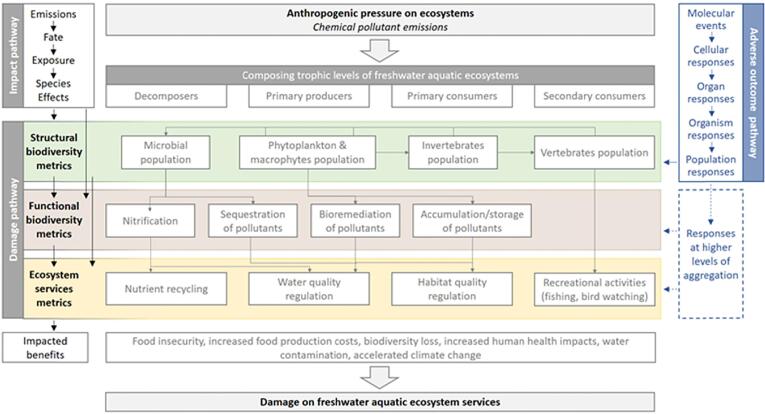
Conceptual framework for translating ecotoxicity impacts into damage on structural biodiversity (e.g. species diversity), functional biodiversity, and freshwater ecosystem services from chemical emissions into the environment. Shown are the steps of a cause-effect chain (left), the current mechanistic reflection of those in Adverse Outcome Pathway approaches (right), and the operational steps utilized in applied ecotoxicology (in the forms of chemical safety assessment and environmental quality assessment). The framework illustrates that various parts are well-developed, whereas other parts are still lacking (dotted box).

In the present study, we illustrate the broader complexity of the impact pathway for freshwater ecosystems and its connections between ecotoxicity, species loss, functional damage, and ES damage. As starting point, we adapted the Adverse Ecosystem Service Pathway (AESP) conceptual framework (Awuah et al., 2020) based on information on the ecotoxicity effects of species food web interactions and ES from Maltby et al.(2021). The principles of that links to other frameworks, especially LCA, but also the Adverse Outcome Pathway (AOP) concept, as all are variants of a causal chain approach, developed and utilized from different perspectives for different practical purposes. Fig. 1 illustrates the overall pathway starting from chemical emissions in different environmental compartments to damage on freshwater ES, whilst relating the various frameworks. The initial step of the pathway, from emission to predicted species-level ecotoxicity effects, commonly yields the Potentially Affected Fraction of species (PAF) exposed by a particular stressor (e.g., a chemical or mixture), as metric of expected impacts resulting from a particular pressure level; for chemical pollutants, this metric is commonly derived from data on across-species differences in sensitivity obtained from laboratory test data for separately tested chemicals. As the thus-predicted impacts empirically relate to effect magnitudes in the field (Posthuma et al., 2020), this metric can empirically be translated into species loss. The functional diversity level further relates ecotoxicity impacts and species loss to damage on freshwater ecosystem functions due to reduction in the performance and characteristics of affected species traits. Finally, species and functional diversity loss is then translated into damage on ES, impacting benefits that humans receive from a well-functioning freshwater ecosystem.

In practice, protection of biodiversity at the ecosystem level still relies primarily on extrapolating ecotoxicity effects at the level of the individual organism. This is based on data from ecotoxicity tests, extrapolated to structural ecosystem properties (i.e., populations and communities) and some ES of importance for human wellbeing, whereby current uncertainties in this assessment process are reflected in the magnitude of uncertainty factors utilized in the derivation of environmental quality standards that aim to ascertain sufficient protection even under data uncertainties (Forbes et al., 2017). However, with the current advancement in mechanistic models and quantitative adverse outcome pathways (AOPs), predictive ecotoxicology is continuously advancing (Forbes et al., 2017, Schmid et al., 2021), such that impacts that may occur when those standards are exceeded are increasingly quantified (e.g., Posthuma et al., 2019).

As shown in Fig. 1, AOPs complement the AESP framework and can be useful for linking ecotoxicity effects to damage on species and functional diversity loss. AOP describes initiating key effects, followed by series of subsequent events, eventually leading to impaired functions in an organism, thereby defining relationships within the AESP concept and providing helpful information in predicting and quantifying impacts up to the community level (Schmid et al., 2021). Although emphasis in the present review is on expanding towards eventual ES damage, the framework in Fig. 1 can be further combined with the Aggregated Exposure Pathway (AEP) concept, which aligns with the overall LCA impact-pathway structure, by allowing to correctly address multiple pathways of exposure that lead to eventual net exposures and eventual damage (Clewell et al., 2020, Escher et al., 2017). To improve the use of AOPs in ecological assessments at a higher level of biological organization, Murphy et al. (2018) proposed a conceptual model linking population models, i.e., the dynamic energy budget (DEB) model and quantitative AOPs, utilizing AOP key events as a measure inducing damage in the DEB variables and processes rates. However, it is still unclear which elements of the AOP concept can be used or adapted as input for quantifying any link from ecotoxicity impacts to damage on ES. More broadly, whilst conceptual approaches and frameworks may be linked as in Fig. 1, their current or future use for decision support also depends on available data.

In the LCA framework, “ecosystem quality” is one of the main defined areas of protection (Verones et al., 2017), with reduced biomass and loss of species richness used to currently indicate damage on ecosystem structure and functioning (Woods et al., 2018). ES are currently addressed at the same level as ecosystem quality and other areas of protection in LCA (Verones et al., 2017). Initial approaches for evaluating damage on ES in the LCA context so far only address land use and land change impact drivers (Liu et al., 2020, Rugani et al., 2019), while linking ecotoxicity impacts to ES damage is currently missing.

The impact pathway from emissions to ecotoxicity impacts expressed at the level of affected species fractions is already covered well in LCA, whereby predicted impacts – expressed as Potentially Affected Fraction of species (PAF) – are extrapolated to damage – expressed as Potentially Disappeared Fraction of species (PDF) on the basis of empirical PAF-PDF associations (Jolliet et al., 2003, Rosenbaum et al., 2008, Fantke et al., 2021). This proxy link, however, requires further refinement to consider relevant differences in impacts when translating those into species loss among species, environments and locations. With that, despite initial attempts, translating predicted ecotoxicity impacts into damage on freshwater species diversity and further to damage on functional diversity and ES is currently not operational in LCA (Liu et al., 2020, Maia de Souza et al., 2018, Othoniel et al., 2015, Rugani et al., 2019, Verones et al., 2017). LCA aims at quantifying the pressure on ecosystems and other aspects attributable exclusively to one or more studied products or system life cycles. With the fact that ecosystems are in reality affected to a multitude of different stressors from all sorts of sources and products, one of the challenges in linking ecotoxicity impacts to damages on ES is to identify which fraction of the damages can be allocated to chemical emissions of a given life cycle. There are, however, motives to develop ES-damage frameworks in LCA, given that currently approx. one-fourth of biodiversity impacts in aquatic ecosystems is attributed to chemical pollution effects (Lemm et al., 2021). In the following, we hence outline a proposal for translating ecotoxicity impacts to species loss, functional diversity loss and finally to damage on ES for consistent inclusion into the LCA framework.

## 3 Source-to-damage modelling approach

Assessing ecotoxicity impacts on freshwater ecosystems requires looking at the source of damage and the overall framework from emission to ecotoxicity damage on species diversity, functional diversity, and ES. The pathway from emissions to ecotoxicity effects is already covered, for instance, in the global scientific consensus model USEtox, where ecotoxicity effects of a chemical emitted into the environment are assessed by combining factors characterizing environmental fate, ecological exposure, and ecotoxicological effects (Fantke et al., 2018, Henderson et al., 2011, Owsianiak et al., 2023, Rosenbaum et al., 2008). Environmental fate factors relate emissions to changes in concentration of a toxicant in the different environmental compartments, including freshwater. Ecological exposure factors then translate the resulting chemical concentrations into the bioavailable fraction of chemicals in the relevant exposure compartments. Effect factors finally link the bioavailable fraction of a chemical in the exposed freshwater environment to impacts on the physiology, behaviour, life history, and ultimately the population of an exposed species (Spurgeon et al., 2020) via different effect mechanisms. This impact pathway commonly ends currently with the quantification of the PDF. Although the potentially disappeared species likely all have their functions in an exposed ecosystem (Faber et al., 2021), the step to damage to functional diversity and ultimately damage on ES delivery still needs to be made.

The scientific literature provides some opportunities that could serve as a starting point for translating ecotoxicity impacts into damage on species diversity, functional diversity, and ecosystem services of freshwater ecosystems in the context of LCA and similar frameworks (see Fig. 1). The opportunities and their features are provided in Table 1. All elements are further elaborated in the subsequent sections.

**Table 1:** Overview of approaches, and their features, that are potentially useful for translating ecotoxicity impacts into damage on species diversity, further relating to damage on functional diversity, and finally linking to damage on ecosystem services of freshwater ecosystems in the context of LCA.

Step	Approach	Description	Data needs/availability	Spatial scope	Assumptions
Ecotoxicity impacts to species diversity damage	The Dynamic Energy Budget (DEB) model models [1]	DEB models explore and predict the effect of a toxicant on both plants and animals growth and reproduction over time and over the entire species lifecycle	Limited data availability	Landscape and regional	Species size is a proxy for species maturity. Processes influencing internal exposure are different from those causing damage
	Food web models e.g., AQUATOX [2]	It represents a full effect on the aquatic food web	Limited data availability	Local and regional	Toxic effect is additive when many organic chemicals are simulated simultaneously
	Population models [3,4,5]	Provide insight into how a toxicant causes stress on individual species population fitness characteristics	Limited data availability	Local and regional	The population is closed demographically and females drive population dynamics
	Mean extinction time [6]	Quantifies the expected survival rate of different species when exposed to a stressor	Limited data availability	Local	No interactions between subpopulations
	Media recovery model [6]	Based on recovering of species richness after exposure to a toxicant	High data availability	Local	The species are assumed to disappear when the toxicant reaches threshold and reappear when the toxicant disappears. The assumption doesn't not hold for a large scale where population reduction would lead to genetic drift and therefore reduction in genetic diversity
	Genetic diversity [6]	Indicates the number of genetically different individuals within the same species	Limited data availability	Local	More genetic variation suggests capacity of the population of organisms to survive stress
	The Principal Response Curve (PRC) approach [7,8]	PRC display effects of a stressor in the course of time	Limited data availability	Local and regional	Follows linearity assumptions but is capable of showing nonlinear treatment effects
	Threshold Indicator Taxa Analysis (TITAN) [9,10]	TITAN approach links field data, to measured environmental concentrations in predicting effects	Limited data availability	Local and regional	Quantitative indices and individual taxon output represent the general nature of community response to a chemical
	Environmental DNA (eDNA) combined with RNA sequencing [11]	Gives an insight into the community composition using the RNA gene expression patterns and the quantity of the DNA	High data availability	Local	A shift in species community composition suggests altered community function
Species diversity damage to functional diversity damage	Trait probability density framework (TPD) [12]	TPD describes the nature of trait distribution within a multidimensional hyper volumes	Limited data availability	Regional	Interspecific variability is considered more significant than intraspecific trait variability
	Functional sensitivity distribution (FSD) [13]	FSD describes the sensitivity of multiple species exposed to a hazardous compound affecting their ecological function	Functional endpoints. Limited data availability	Local	FSD of tested species resembles the FSD of species assemblage in the field
	Phenotypic diversity model [6]	Links directly phenotypic variation to ecosystem functioning	Limited data availability	Local	Reduction in phenotypic variance from toxic pressure affects ecosystem functioning
Functional diversity damage to ecosystem services damage	Common International Classification of Ecosystem Services (CICES) [14,15]	Hierarchical classification system which is tailored to accounting i.e., the value of ecosystems and the cost of their depletion taking into account abiotic resources	High data needs	Local and regional	Focuses on identification of the final ES directly linked to values valued by human beings
	National Ecosystem Services Classification System (NESCS) [14,15]	Hierarchical classification system which identifies pathway through which changes in the ecosystems impact ES flow to humans	High data needs	National	There is a clear division between natural systems and human systems
	Final Ecosystem Goods and Services Classification System (FEGS-CS) [14,16]	Hierarchical ES classification framework that provides distinction between intermediate and final ES and linkage between ES flow and human well being	High data needs	Local and regional	There is a fine separation of the intermediate and final ES
	Cascade model [17]	Represents the flow of ES in a logical scheme of chains from their generation to their value to humans well-being	High data needs	National	_ES flow in a linear, logical scheme of chains_
	Ecological Production Functions (EPFs) [18]	Quantifies connection between ecosystem structure and processes to ecosystem function and ES importance for human wellbeing based on function –related descriptors	High data needs	Local	EPFs represent outcomes of ecological processes

[1: EFSA et al., 2018], [2: Park et al., 2008], [3: Earl, 2019, 4: Forbes et al., 2017, 5: Maltby et al., 2021], [6: Larsen & Hauschild, 2007], [7: Van Den Brink et al., 2000, 8: Moser et al., 2007], [9: Berger et al., 2016, 10: Baker & King, 2010], [11: Birrer et al., 2021], [12: Carmona et al., 2016], [13: Posthuma & de Zwart, 2014], [14: Maia de Souza et al., 2018, 15: US-EPA, 2018], [16: Landers & Nahlik, 2013], [17: Rugani et al., 2019], [18: Faber et al., 2021].

### 3.1 From freshwater ecotoxicity to damage on structural species diversity

Effects of chemicals on freshwater ecosystem species range from direct acute and chronic toxicity in organisms to many sub-lethal or indirect impacts on behaviour, functional roles, predator–prey relationships, and food web dynamics (Chagnon et al., 2015). If considered mechanistically, assessments would require quantification and understanding of the full set of linkages between direct ecotoxicity effects and their consequential damage if they should be translated into species loss and associated changes in food webs, functions and services. Various elements of this ‘full approach’ have received attention, to be potentially developed into practicable approaches.

Three approaches were initially developed to be potentially used as a starting point to translate ecotoxicity impacts into damage on species diversity expressed as species loss. These approaches include the media recovery approach that is based on species richness (the number of individuals or biomass) recovery after exposure to a toxicant, the mean extinction approach that quantifies the expected survival rate of different species when exposed to a stressor, and the genetic diversity approach that is based on changes in species genetic diversity (Larsen & Hauschild, 2007). The genetic diversity approach could help solve problems with addressing diversity within species versus diversity between species (the latter is what we refer to as 'species diversity'), focusing on within species and between population variations.

Genetic and species diversity are fundamental components of assessing impacts on biodiversity (Hoban et al. 2022). Both are influenced by the same ecological processes: species selection, migration, drift, and speciation/mutation (Vellend, 2010). Genetic diversity, that is variation in the genetic make-up of species, enables populations to adapt to changing environments and offers ‘insurance’ against stressor impacts (Vellend & Geber, 2005), such that individuals with desirable traits (i.e., alleles) in a population can survive to produce offspring and allow for the continuation of generations. In contrast, species diversity focuses on variation between species, i.e., the number of species within a community (Vellend & Geber, 2005).

The possibility that genetic and species diversity influence each other has been acknowledged for decades (Bolin & Lau, 2022). A positive relationship between species diversity and genetic diversity has been observed in communities exposed to certain stressors (Vellend and Geber, 2005, Blum et al., 2012). This positive relationship can be linked to the genotypes of a focal species having a competitive advantage against different species within the community, and other species having a competitive advantage against the genotype of common focal species (Bolin and Lau, 2022, Vellend, 2006, Vellend, 2008). However, high genetic diversity can also negatively influence species diversity if it reduces available niche spaces for heterospecific species (Bolin and Lau, 2022, Vellend and Geber, 2005). In some cases, genetic diversity may change without a change in species abundance (Hoban et al., 2022), while changes in species diversity may alter the positive species interactions resulting in changes in the ecosystem processes (Cardinale et al., 2002). However, these approaches are currently rarely used, mainly due to their intrinsic complexity and low availability of data, especially for the mean extinction and genetic diversity approaches (Larsen & Hauschild, 2007). Environmental DNA (eDNA) describes the use of species DNA extracted from soil, water, or ice. Combined with gene sequencing, eDNA provides a way of measuring species diversity, assigning functionality, and consequently gaining an insight into food webs without species observation or trapping (Birrer et al., 2021). However, it is difficult to accurately quantify species diversity from eDNA, since different species shed DNA at different rates, which is also influenced by environmental factors such as UV light and microbial activity (Goldberg et al., 2016). Thus, due to DNA degradation, only the recent presence of species can be accurately detected (Goldberg et al., 2016, Rees et al., 2014).

Another type of approaches in linking ecotoxicity effect to species loss (i.e. loss in species diversity) consists of the idea to develop and use mechanistic models such as dynamic energy budget models (DEB), population models, and food web models to extrapolate effects at individual species levels to damage at the population level or community level (Faber et al., 2019, Forbes and Galic, 2016, Forbes et al., 2017). DEB models simulate how species assimilate and allocate energy for physiological processes (e.g., growth, development, and reproduction) while also reflecting how changes in the environmental conditions (e.g., exposure to chemicals, resource availability, and temperature) change those energy flows (Dong et al., 2022, Forbes et al., 2017). DEB models facilitate extrapolation of chemical effects across species and service providing units (Forbes et al., 2017). DEB models are also flexible, allowing for incorporation of chemical modes of action depending on the processes affected by the toxicant. Thus, they provide a potential to mechanistically explore toxicity beyond mere dose effect descriptions for separate ecotoxicity endpoints (EFSA et al., 2018). However, DEB models are compound- and species-specific, with currently only a very limited array of species and chemicals covered (EFSA et al., 2018).

Population models are another opportunity, which utilizes information on individual species' life history characteristics (such as juvenile period, growth rate, reproductive output), thus bringing additional biological realism when predicting damage to populations from data on various endpoints (Forbes et al., 2017, Maltby et al., 2021). However, population models extrapolate changes in specific individual species performance to impacts on population dynamics and structure, with a need to cover a broader range of species (i.e. limited number of possible species for which models are readily available) and flexibility in predicting ecotoxicity effect under different conditions and habitats (Ockleford et al., 2018, Maltby et al., 2021).

Food web models, such as AQUATOX (Park et al., 2008), consider the flow of toxic substances through the food web (i.e., species interactions) and ecotoxicity impacts on the food web structure (Faber et al., 2019, Maltby et al., 2021). Thus, food web models would provide the damage information aimed at, when it is known which species are threatened by the presence of a toxic substance and how that affects the food web structure and/or function (Jørgensen, 2016). Food web models can provide information on the biomass of species, individuals, and populations with a possibility to further predict damage on ES (Galic et al., 2019). However, food web models have not yet been widely used because of the difficulty of modelling the flow and fate of toxic substances in complex and highly spatiotemporally varying food webs (Jørgensen, 2016). Food web models like AQUATOX can currently model effects associated only with organic chemicals (Park et al., 2008). Furthermore, the lack of standardized impact indicators currently limits the applicability of food web models for use in practical LCA (Maltby et al., 2021).

Translating ecotoxicity impacts into species loss can also be achieved using the principal response curve (PRC) approach. This approach uses data on multiple species responses from controlled experiments, e.g., mesocosms. However, PRC statistics are only feasible for data with repeated measures over time (Van Den Brink et al., 2000, Van Den Brink and Braak, 1999). Unlike mechanistic models that allow for extrapolation of ecotoxicity effects to novel conditions, the PRC approach can usually not be extrapolated beyond experimental test conditions (Jager, 2016, Forbes et al., 2017). Furthermore, it is not possible to recognize sensitive species with a different response pattern with the PRC method (Moser et al., 2007).

In contrast to PRC derived from mesocosm-type test data series, the Threshold Indicator Taxa Analysis (TITAN) approach uses field monitoring data on multiple stressed system to derive species-specific differences in abundance response thresholds given pressure level gradients (Baker & King, 2010). TITAN's capacity to identify abrupt changes (so-called “breaking points”) in occurrence and abundance of taxa along a chemical gradient makes it appropriate to identify sensitive taxa showing a clear response to a chemical gradient under field conditions (Berger et al., 2016). Given that TITAN analyses can be used to track changes in species abundance under chemical pollution pressure, in terms of fractions of species affected at given field exposures (Berger et al., 2016, Baker and King, 2010), there is latitude to use TITAN to characterize field effects across species, and relate that to the predicted impacts as generated with SSD models. With that, the TITAN approach is a promising empirical starting point for relating predicted ecotoxicity impacts (PAF) into damage in the field in terms of species loss (PDF). However, the approach is constrained by limited data availability, i.e., to be operationally applied in the LCA framework, it requires large-scale monitoring data with species occurrences and abundance patterns at different sites along with measured chemicals or mixture concentrations. That is, the use of the TITAN approach provides insights in empirical PAF-PDF associations for particular study areas, particular chemical pollution pressures and particular species groups, so that LCA damage assessment would be best served by analysis of diverse, multiple field response data sets. As yet, available work consist of (Berger et al., 2016) analyses, and ongoing work focuses on establishing PAF-PDF relationships for Dutch surface water monitoring data.

The challenges of most mechanistic models and the empirical approaches are partly conceptual but mostly also related to available data, as highlighted above, including the need to cover a wider variety of species, currently limited coverage of chemicals and different organisms' specific endpoints, which still require attention. Using the SSD approach to cover a broader range of species can bridge part of the data-related gap and with that can help refining some of the models (EFSA et al., 2018). Furthermore, comparing the magnitude of different effect endpoints (e.g. reproduction vs growth) from SSDs would provide an option of deriving consistent metrics for translating ecotoxicity effects into damage at species diversity level while utilizing available data.

### 3.2 From species loss to damage on functional diversity

Functional diversity is the variation of traits between organisms (Carmona et al., 2016). Species' functional traits determine how they respond to environmental conditions and disturbances, such as emissions of chemical stressors. Characterization of functional diversity through various components such as functional richness, functional evenness, and functional divergence has great potential to answer different ecological questions, including impacts of any disturbance on the assembly of biological communities. Functional evenness is the amount of functional volume occupied by a trait density distribution indicating a range in a single trait case. Functional richness is the amount of space occupied by species in an ecological unit. In contrast, functional divergence is an indicator of the degree of the distribution of abundance within the functional trait volume (Carmona et al., 2016).

At the community level, estimating functional diversity within a community of species is often determined as a function of differences in individual species traits (Carmona et al., 2016). That is, any stressor that has a strong influence on the composition and diversity of species traits and interaction in the food web is having an influence on an ecosystem function based on those traits (Truchy et al., 2015, Faber et al., 2019, Maltby et al., 2017a, Maltby et al., 2017b).

Ecosystem functioning relates to the sum of all processes that sustain an ecosystem through biological activities (Reiss et al., 2009, Truchy et al., 2015). Processes at the ecosystem level emerge from species' interaction with each other in their food web and with the environment, which often involves transformation of nutrients and energy, generation of the species habitat structures, and maintenance of the species populations (Truchy et al., 2015, Faber et al., 2019, Maltby et al., 2017a, Maltby et al., 2017b). Dominant processes associated with freshwater ecosystem functioning are nutrient cycling, organic matter transformation, primary productivity, secondary productivity, and ecosystem metabolism (Harrison et al., 2022). A specific process consists of the option of sequestration or detoxification of pollutants influencing water quality in the ecosystem (Maltby et al., 2021). As discussed in Haines-Young & Potschin (2010), ecosystem functioning is highly associated with species biodiversity, such that a decrease in ecosystem functioning occurs more rapidly when there is low species diversity. Apart from the number of different species (i.e., species diversity), other measures of biodiversity essential for ecosystem functioning include species abundance, the composition of the genotypes in the ecosystem population, and functional groups (Haines-Young & Potschin, 2010). As much as an ecosystem can reduce species diversity without impacting its functioning due to redundancy in species' functional traits, the redundancy of functional groups ensures a continuous functioning of an ecosystem (Baumgärtner, 2007). Such redundancy largely depends on the presence and composition of species functional groups and traits (Faber et al., 2019, Haines-Young and Potschin, 2010, Rumschlag et al., 2020).

Chemical pollution may have a specific impact in ecosystems and their functional characteristics. That is, differences in the match, or mismatch, of chemical modes of action and species traits (e.g., insecticides and insect traits presence or absent) determine how chemical exposures affect species and which consequences on ecosystem functioning or to be expected (Chagnon et al., 2015). Chemical modes of action can also help identify the most sensitive species. That is, such a species or set of species traits may form the food web, so that the entire functioning of the ecosystem would be compromised if the sensitive species are affected, much more than when the sensitive species are at the end of the food web. For example, exposure of phytoplankton to herbicides decreases community composition before a decline in ecosystem functioning, i.e., reduced community respiration and primary productivity (Rumschlag et al., 2020). In contrast, insecticides reduce zooplankton composition before impacting community respiration and the primary productivity of phytoplankton (Rumschlag et al., 2020).

According to Sodré & Bozelli (2019), chemical stressors can decrease organisms' body size, thus affecting many physiological functions. The magnitude of a biotic ecosystem function is a consequence of the rate of ecosystem processes and related change in producing biomass (e.g. photosynthetic rate and primary producers' biomass). Considering ecosystem functions takes into account the number of species (richness), identity (composition), and abundance of species in a community that contribute to a specific function.

The function sensitivity distribution (FSD) approach has been proposed to quantify the impact of a toxic chemical on the functioning of an ecosystem by considering function-related endpoints (Posthuma et al., 2001). Its application would be based on the empirical observation that – similar to differences across species in sensitivity to chemical exposures – the functional endpoints follow a bell-shaped distribution. Development and application of FSDs would enable direct evaluation of a functional damage assessment, similar to the establishment of the PAF-PDF relationship which can be determined utilizing TITAN analysis, as described above. However, this approach is currently rarely used due to its limited data availability (Posthuma & de Zwart, 2014).

Given various concepts and components in estimating functional diversity, Carmona et al. (2016) proposed a trait probability density (TPD) framework that unifies existing quantification approaches for functional diversity components. TPD considers species abundance and intraspecific trait variability to derive estimates for different functional diversity components, i.e., functional richness, functional evenness, and functional divergence. With available data, using TPD would, allow predictions of functional impacts across various spatial scales, given that it is assumed that values of the TPD framework of an ecological unit are directly proportional to the relative abundance of their trait values (Carmona et al., 2016). TPD functions may be directly applied to predict the functional structure of species populations and communities along chemical gradients. The method requires substantial trait data (Carmona et al., 2016).

The phenotypic diversity model (i.e., genetic relationship between different groups of species) could also provide a way to translate changes in species diversity into damage on ecosystem functioning. Species diversity directly links phenotypic variance to ecosystem functioning, represented as a change in biomass production in an ecosystem from a toxic pressure. With a focus on species functional groups as the basic unit of the ecosystem, species sensitivity is taken into consideration in this approach (Larsen & Hauschild, 2007).

Functional indicators that measure functional effect traits or rates or attributes of processes have been proposed. Such indicators have been proposed, since it is considered difficult to measure ecosystem functions or predict them from underlying structural impacts. On this relationship, it can be reasoned that highly aggregated functional metrics (such as primary productivity) are relatively insensitive as compared to underlying structural impacts. Exploiting the relationship between potential functional indicators that are more directly connected to mechanistic processes can help link species loss to ecosystem function loss by assessing how a change in the state related to processes impact rates of processes within the food web. However, changes in multiple interacting functions at the food web level and across different trophic levels are indicated by processes measured at the food web level, such as the flow of energy through the food web (Harrison et al., 2022).

Combining different functional diversity components, FSD, and functional indicators (Posthuma and de Zwart, 2014, Carmona et al., 2016, Harrison et al., 2022) can hence provide a possible starting point in translating species loss to damage on functional diversity. Furthermore, eDNA and sRNA measurements may provide a direct way of measuring species diversity, in addition to getting an insight into the community function dynamics from direct observation of species (biomonitoring data).

An overview of the features of different approaches that could potentially serve as a starting point for translating damage on species diversity into damage on functional diversity of freshwater ecosystems in the context of LCA is provided in Table 1. Different functional indicators with related taxa and processes are provided in Table 2, for metrics representing rather high levels of aggregation.

**Table 2:** Functional indicators possible for translating species loss to damage on ecosystem functioning with related taxa and processes dominant for freshwater ecosystem (Harrison et al., 2022).

Ecosystem function	Processes	State related to processes	Freshwater taxa	Food web metrics
Ecosystem metabolism	Respiration, extracellular enzyme activity, amino acid uptake in biofilm, microbial electron transport system activity	Dissolved oxygen concentration	Microbes	Substrate use metabolic profile
Organic matter transformation	Leaf litter decomposition, detritivores feeding rate	Biomass of fungi	Fungi, invertebrates detritivores, heterotrophic microbes	Detritivores feeding preference
Nutrient cycling	Denitrification, Nitrogen dioxide flux	Total P or C or N; Organic C or N; Nitrites or Nitrates	Microbes	Functional composition and traits of taxa
Primary productivity	Rates of biomass production, oxygen production or carbon dioxide consumption	Biomass or abundance or density of algae, biofilm, phytoplankton, or macrophytes Chlorophyll-a concentration, amount of glutamine sythetase	Macrophytes, algae, phytoplankton, autotrophic microbes	Fish functional composition, invertebrates feeding groups
Secondary productivity	Growth rates or rates of biomass production	Biomass or abundance or density of heterotrophic microbes, invertebrates, or fish	Vertebrates, invertebrates	Phytoplankton functional composition

### 3.3 From functional loss to damage on ecosystem services

Damage on functional diversity loss can be linked to damage on related ES as an intermediate step of the main pathway in linking ecotoxicity effects to damage on ES (Truchy et al., 2015, Maltby et al., 2021). However, there is also a direct link from species loss to damage on ES, without explicitly considering the intermediate step of evaluating affecting any function (Maltby et al., 2021).

Freshwater ES are dependent on freshwater organism interactions and processes (Chagnon et al., 2015). For example, microbial decomposers and invertebrate detritivores degrade leaf litter, which in turn aids in nutrient cycling. However, when microbial decomposers and invertebrate detritivores are exposed to toxic chemicals, it may cause feeding inhibition and mortality. This, in turn, might damage ecosystem services such as leaf litter breakdown, decomposition, and primary productivity rate and flow of ES, e.g., nutrient cycling and support for other freshwater organisms (Peters et al., 2013, Chagnon et al., 2015).

Biodiversity is the variety of life forms, including the variation of genes, species, and functional traits. Biodiversity and ecosystem functioning relationships (BEF) have been studied for several decades (Cardinale et al., 2012, van der Plas, 2019), with researchers often reporting the BEF relationship as nonlinear. Diversity of the community positively influences ecosystem functioning (van der Plas, 2019). While biodiversity loss reduces the number of genes, species, and functional groups, it consequently decreases the efficiency by which species communities capture essential resources, produce biomass, decompose and recycle nutrients (Cardinale et al., 2012).

Some studies have shown that environmental change may damage ecosystem functioning without affecting species richness by affecting population density and community composition as the community competes for limited resources at one trophic level (Spaak et al., 2017). However, biodiversity loss across trophic levels can influence ecosystem functioning more strongly than diversity loss within a trophic level, since food web interactions are key mediators of ecosystem functioning (Cardinale et al., 2012). Hence, high biodiversity is required to maintain the multifunctionality of ecosystems across spatial and temporal scales (Cardinale et al., 2012).

BEF has often been measured without extending to known ES. Likewise, biodiversity and ecosystem services relationships (BES) have often been described without understanding the underlying ecosystem functions (Cardinale et al., 2012). Predicting biodiversity-related consequences on ES also requires understanding of which functional traits place biodiversity at a higher probability of extinction or establishment, i.e., response traits, and how response traits drive ecosystem functioning, i.e., effect traits (Cardinale et al., 2012, Suding et al., 2008).

For example, diverse communities are more productive because they contain key species that greatly influence productivity, and differences in functional traits increase the total resource capture (Cardinale et al., 2012). Furthermore, functional traits influence the extent to which ecosystem functioning changes after the extinction of biological traits (Cardinale et al., 2012).

Many ES ultimately depend on the variety of life forms (Scherer-Lorenzen et al., 2022). Therefore, successfully understanding the linkages between biodiversity, ecosystem functioning and ES requires quantifying the networks of mechanistic links between ecosystem functions and ES using e.g. mechanistic models (Cardinale et al., 2012). However, challenges still exist when incorporating ES regulated by multiple functions in the BEF relationship, which does not necessarily respond to changes in biodiversity in the same way. Mismatch in how organisms interact at different spatial and temporal scales also complicates integrating food webs into BEF and BES (Cardinale et al., 2012).

According to van der Plas (2019), functional diversity is a stronger predictor of ecosystem functioning than biodiversity, partly because of the presence of a particular functional group (i.e., keystone species) that drives ecosystem processes or abiotic conditions that outweigh the biodiversity effect, such that environment variation and biodiversity jointly drive ecosystem functioning.

Studies directly assessing ecotoxicity impacts on freshwater ecosystem functioning, which could facilitate further translation of functional loss to damage on ES, are rare due to little understanding of biodiversity-ecosystem-function/services relationships and the availability of mechanistic models (e.g., ecological production functions, EPFs) to link chemical-induced effects on individual species to ES delivery (Faber et al., 2019).

The quantitative ecological production functions (EPFs) approach provides quantifiable links from ecosystem functional diversity loss to damage on ES flows (Faber et al., 2019) or a direct link of ecosystem characteristics (i.e., SPU) to final ES (Bruins et al., 2017, Forbes et al., 2017), which can be used as a starting point for translating species loss into damage on ES. Online models, such as U.S. Environmental Protection Agency EcoService, have been developed based on the EPFs approach to quantify damage on ES (US EPA, 2018). However, no standardized test exists for most taxa in EPFs (Faber et al., 2021). Also, existing quantitative models incorporating ecological production functions have limited chemical exposure dose–response relationships (Faber et al., 2019), which are essential as they can be further extrapolated to damage on related ES.

Syberg et al. (2017) proposed to create a 'direct' link from ecotoxicity impacts (using PAF as predicted impact metric) to damage on ES until the full pathway from ecotoxicity impacts via damage on genetic and function diversity to damage on ES is better understood. In the approach proposed by Syberg et al. (2017), damage on ES from ecotoxicity impacts is derived from the sum of hazard quotients (HQ) across chemicals i that is derived as ratio of measured chemical concentrations in freshwater environments ($C_{i}$, mg/l) and the related threshold ($C_{\mathrm{ref},i}$, mg/l) set to indicate an upper-limit safe chemical level for human consumption for each chemical as $\mathrm{HQ}=\sum_{i}\left(C_{i}/C_{\mathrm{ref},i}\right)$. This approach can be considered a pragmatic approach which sets a human health related upper boundary on chemical exposure, such that exposure of man through ecosystems is not affected by separate chemicals or unintended mixtures, whilst exceedance of that boundary would warrant remediation to safeguard human health.

ES conceptual frameworks also offer ways of linking ecosystem functioning loss to damage on the ES. From earlier reviews conducted on ES methods and applications to freshwater ecosystems (Bagstad et al., 2013, Maia de Souza et al., 2018), most established methods, such as the InVEST approach, help assess risk from land use change or climate change, but applications in response to chemical stressors have not been studied. Maia de Souza et al. (2018) suggest applying NESCS and FEGS-CS, ES classification frameworks to understand the impacts between ecosystem functions and final ES provided for humans, which could also serve as a starting point for application in the LCA framework. FEGS-CS and NESCS frameworks can translate damage on the functional level of an ecosystem to damage on ES and offer a distinction between intermediate and final ES (Maia de Souza et al., 2018). Intermediate ES are not directly used or consumed by humans but are considered necessary for producing final ES delivery.

The cascade model proposed by Rugani et al., 2019, Liu et al., 2020 links changes in ecosystem structure and functions to human wellbeing changes in a cause-effect chain model in soil ecosystems. With that, this model complements the LCIA impact-pathway framework by providing information about trade-offs (i.e., costs and benefits) of a particular stressor on ES flows (Rugani et al., 2019). However, ecotoxicity-related aspects and their influence on freshwater ES are not currently addressed in the cascade framework. In addition, this model is currently not able to address the dynamics and nonlinear nature of ES (Maia de Souza et al., 2018).

Overall, numerous knowledge gaps remain for successfully translating ecotoxicity impacts into damage on freshwater ES, either directly from species loss or through functional diversity loss. This includes (a) the lack of comprehensive and integrated approaches to assess impacts of chemicals and other stressors while taking into account different routes of chemical exposures, (b) the overestimation or underestimation of potential chemical risk on SPUs, which reduces the accuracy of ES assessment, (c) the complexity in analysing ES trade-offs, i.e. protecting one ES resulting in downstream effects on other ES (Syberg et al., 2017).

The challenge of overestimation or underestimation of the risk on SPUs may be addressed in part by generating separate SSDs for different species groups, which uses ecological information on species communities such as functional groups or trait characteristics (Van den Brink et al., 2021). This may help identify SPUs, i.e. ES that are potentially at risk (Faber et al., 2021, Oginah et al., 2021).

Current methods that link individual elements along the pathway from ecotoxicity impacts to damage on ES delivery (Fig. 1) are still in their infancy, and possible adaptations are in the early stages. Current methods or frameworks do not systematically link ecosystem functions loss to damage on ES from chemical impacts. However, applying the ES frameworks and cascade model, which incorporates EPFs, provides a possible way forward to translate functional loss to damage on ES and with that to include damage on ES associated with ecotoxicity impacts on freshwater ecosystems into LCA. The aggregated ES consequences resemble the aggregated life cycle impacts in terms of species losses modeled at damage level in current state of the art LCIA methods. It is not the intention, however, to predict concrete ES consequences in any specific ecosystem but rather to estimate an overall consequence of a given product or system life cycle.

## 4 Monitoring-based framework for ES assessment and management

One of the key problems of ecotoxicity assessments and assessing damage is the need for laboratory-to-field extrapolation, given that stressors studied in applied ecology (such as nutrient enrichment) are addressed based on ecological concepts and field data, whilst stressors studied in applied ecotoxicology are most often relaying on laboratory toxicity data. Whilst there are mechanism-based approaches which could be applied in PAF-PDF characterization of damage, it is key to highlight the final issue that the predicted damage should relate to true damage, that is: that the lab-field extrapolation for chemical pollution impacts is correct. The latter can be judged by analyses of landscape-level ecosystem data. Assessment and management of ES eventually require data-driven insights to recognize ES deterioration upon adding more man-made pressures and improvement upon less man-made pressures. Data-driven insights can be obtained from (bio-)monitoring data, combined with appropriate statistical analyses. The latter should be able to characterize the relative roles of different pressures on ecological metrics, be it species abundance data, aggregated structural biodiversity metrics, or aggregated ES metrics. In an ideal case, the damage predicted by any of the mechanistic models should relate to damage in the field.

Generally, the (bio-)monitoring data should cover a number of sites that vastly exceed the number of pressure metrics to avoid the so-called 'curse of dimensionality’. Few sites mean that each added pressure parameter reduces the power of statistical analyses unless sufficient increases in the number of study sites are substantiated. One of the key problems in this respect is the study of chemical pollution through separate exposure or risk metrics for each chemical. The problem was solved by summarizing all chemicals, or mode-of-action subgroups, via mixture toxic pressure quantification (Posthuma et al., 2019).

The statistical diagnostic assessments also need to take into account that there are different types of ecosystems (e.g., a lake, a river, a brook), such that the natural conditions are represented in a multitude of non– or minimally disturbed ecosystem types, whereby damage should be considered relative to those different reference states.

Regarding the statistical analyses aimed at diagnosing relationships between pressure variables and impact variables, the best 'training' data need to consist of the longest possible data gradients for all pressures (e.g., very low to very high pH, ibidem toxic pressure), where the covariance amongst the pressures is below a critical level. This can be checked by calculating, e.g., the Variance Inflation Factor, which should be below a threshold above which interpretation bias (in diagnosing probable causes of impacts) occurs (Lemm et al., 2021).

Monitoring-based approaches involve repetitive data collection to determine trends in parameters or endpoints that comprise ES (Chapman, 2012). Characterization of spatial and temporal relationships and trends in (bio-)monitoring data, aimed at relating multiple pressures to variation and changes in biotic parameters, can assist in predicting the future status of ES under alternative management strategies. At the global level, the Group on Earth Observations Biodiversity Network (GEO BON ES) was established to promote the monitoring of biodiversity and ecosystems for the scientific community and decision-making (Vaz et al., 2021). With satellite sensors, aspects of ecosystem functioning, such as the primary production, can be quantified (Vaz et al., 2021).

Multiple stress analyses have been made for various pressure combinations, areas, species groups, and practical aims. Examples are Grizzetti et al., 2019, Lemm et al., 2021, focusing on characterizing water quality as a function of a suite of pressures, including unintended complex mixtures. The examples are suitable for exploring and prioritizing alternative management scenarios' potential effects. Similar studies exploring such matters for ES are scarce.

There are global monitoring platform for ES and biodiversity inspired initiatives, such as the Global Biodiversity Information Facility (GBIF) and the Ocean Biodiversity Information System (OBIS) (Vaz et al., 2021). These approaches still face challenges, such as the lack of methods to combine ES monitoring observations and data across different scales, harmonized ES metrics that link interactions between people and ecosystems, and difficulty in incorporating diverse social-cultural values and knowledge into monitoring activities (Vaz et al., 2021). All those problems have been recognized in the diagnostic studies of non-ES impact metrics, confirming that successful studies require a combination of sufficient site numbers (given pressure numbers), good handling of natural variability of non– or minimally disturbed ecosystem types, and a sufficiently wide range of non– or limitedly co-varying pressure metrics, whilst recognizing the specific situation for chemical pollution (and the laboratory-field extrapolation issue) as pressure factor.

In the ES field, monitoring can have a different focus. For instance, for recreation fishing ES, monitoring can either focus on the effect of a stressor on the fishery SPU values, on the ways of preserving fishery SPU values, and on the state of the ecosystem in terms of the SPU, i.e., effect-based monitoring (Chapman, 2012). Because the effects may be incorrectly attributed to the measured chemicals when focusing on those separately from the other pressures, multiple stressor analysis is recommended as a better way of monitoring damage on ES (Chapman, 2012). An example is monitoring toxic pressure across the Netherlands on water quality (KIWK, 2022). This study calculates the key toxicity factor from previous water quality information, such as contaminant locations, causes and measures taken. Water quality managers use the key toxicity factor as a decision-support tool to identify locations and substance groups that most threaten the water quality (KIWK, 2022).

An attempt was also made earlier to monitor the ecological status of the aquatic ecosystem in Europe as an indicator of water quality, which involved using ecological status metrics from biological quality elements information instead of raw field monitoring data (Posthuma et al., 2020). Using the biological quality elements was a key step that solved the issue of natural differences in non– or minimally disturbed reference status across ecosystems. Because current knowledge on monitoring freshwater ES and stressors is usually stored on separate data platforms, without spatial alignment, it is currently not straightforward to execute a diagnostic analysis of ES data at any geographical scale, apart from some early studies such as (Grizzetti et al., 2019).

For a holistic understanding of how ES can be influenced by one or multiple man-made pressures, efforts are still needed to further develop the data, statistical analysis frameworks, and tools that combine knowledge of ES monitoring with the status and trends of stressors at different spatial and temporal scales. This is particularly challenging when there is interest in chemical pollution as a spatio-temporally variable pressure next to various other pressures, given that applied ecology and applied ecotoxicology need to be bridged by summary concepts such as 'mixture toxic pressure.'.

## 5 Conclusions and outlook

To address damage on freshwater ES in LCA associated with toxic chemical emissions along product and technology life cycles, related ecotoxicity impacts need to be linked to damage on species (i.e. structural) and functional diversity and finally to damage on ES. This needs to consider approaches that utilize field-based monitoring data with biological realism and align with LCA boundary conditions.

For a holistic assessment of the entire ecosystem rather than individual species population, models that consider multiple populations or entire food webs (Jørgensen, 2016) can help translating ecotoxicity effects into species loss, expressing damage on an ecosystem's species diversity. However, because such models depend on extrapolation of effects to higher biological organizations, leading to higher uncertainty in the output, a novel approach such as TITAN is a promising way forward, which instead builds on field-based monitoring data. TITAN approach, however, has high data needs that are currently available for a few study areas, specific pressure sets and specific taxonomic groups under study.

A trait probability density framework incorporating various functional diversity components can subsequently link species loss to functional diversity loss. However, more data with functional diversity endpoints are still needed before this framework can be operationalized.

Quantitative ecological production functions could finally translate damage on species diversity to functional loss and damage on ES, if uncertainty in extrapolating from the relevant SPUs and functions to ES is considered (Maltby et al., 2021). The challenge of multiple chains of effects can be potentially addressed by applying population or food web models to identify the structural changes in the food web due to the direct or indirect impact of a chemical or other stressor (Maltby et al., 2021). However, there is a need to develop robust models that extrapolate chemical-induced changes in key SPU attributes to changes in ES delivery by incorporating knowledge on how SSDs can be reliably used to address effects on specific species groups associated with certain ES over other species groups that are less affected i.e. split-SSDs (Maltby et al., 2005, Van Den Brink et al., 2006, Maltby et al., 2009) and including EPF that integrate multiple ES and their potential interactions.

The advantage of using EPF-based approaches is that they allow for measured functional endpoints to be further linked to changes in ES delivery. However, identifying endpoints suitable for ecosystem assessment remains a challenge (Syberg et al., 2017), where for example additional functional endpoints should be considered that are particularly relevant for freshwater ecosystems (Maltby et al., 2017a, Maltby et al., 2017b, Faber et al., 2021). At the global levels, frameworks or tools that may combine knowledge of ES monitoring and status and trends of chemical and other stressor at different spatial and temporal scales are still needed. The ideal-world expectation for decision support would provide the assessor with specific damage insights per region; however, LCA is an approach founded in the emitter-perspective, which delivers generic potentials to cause harm also useful for decision support purposes. The outputs of LCA are useful as they allow for generically selecting the least-harmful, functionally equivalent product systems.

Overall, we highlighted key elements to develop a framework and associated potentially useful approaches for integration in LCA and similar assessment frameworks that link ecotoxicity impacts on aquatic freshwater species to damage on genetic and functional diversity at the ecosystem level, and further to damage on ES delivery. More attention needs to be paid to developing and refining mechanistic damage models with standardized functional endpoints and structures that align with cause-effect chain modelling, such as the cascade model. By providing an overall framework as well as an evaluation of potentially useful scientific and practical approaches, our study constitutes a useful starting point for addressing current challenges in linking ecotoxicity impacts to damage on freshwater ES, either directly from species loss or through functional diversity loss.

## Declaration of Competing Interest

The authors declare that they have no known competing financial interests or personal relationships that could have appeared to influence the work reported in this paper.

## Data Availability

No data was used for the research described in the article.
